# Selenium nanoparticles promotes intestinal development in broilers by inhibiting intestinal inflammation and NLRP3 signaling pathway compared with other selenium sources

**DOI:** 10.1016/j.psj.2024.103958

**Published:** 2024-06-07

**Authors:** Yanhong Chen, Caiwei Luo, Shu Li, Xingbo Liu, Yanbing Guo, Yuxin Li, Yuanzhi Wang, Jianmin Yuan

**Affiliations:** ⁎ State Key Laboratory of Animal Nutrition, College of Animal Science and Technology, China Agricultural University, Beijing 100193, China; † College of Resources and Environmental Sciences, China Agricultural University, Beijing 100193, China; ‡ Microbiology and Immunology Department of Preventive Veterinary Medicine, College of Veterinary Medicine, China Agricultural University, Beijing 100193, China

**Keywords:** broiler intestine, goblet cell, Nano-Se, NLRP3 signaling pathway, SeMet

## Abstract

This study aimed to investigate how various selenium sources affect the intestinal health of broiler chickens. A total of 384, one-day-old Arbor Acres broilers were weighed and randomly allocated to four treatment groups. The control diet was a basal diet added with: 0.2 mg/kg Sodium Selenite (**SS**-control), 0.2 mg/kg Selenium nano-particles (**Nano-Se**), 0.2 mg/kg Selenomethionine (**SeMet**), and 0.2 mg/kg Selenocysteine (**Sec**) as the treatments. The results indicated that Nano-Se and SeMet were effective in enhancing the villus height (**VH**) and the villus height/crypt depth ratio (**VH/CD**) in the jejunum compared to (**SS**) (*P* < 0.05). The inclusion of Nano-Se into the diets increased the mRNA levels of zonula occluden-1 (***ZO-1***), *ZO-2, Occludin, Claudin-1*, and *Claudin-3* compared to the SS diet (*P* < 0.05). The SeMet increased the levels of *ZO-1* and *Claudin-3* compared to the SS (*P* < 0.05). Moreover, SeMet upregulated the marker genes of intestinal enteroendocrine cells, stem cells, and epithelial cells compared to the SS diet (*P* < 0.05). However, supplementation of Nano-Se reduced the mRNA levels of interleukin 1β (***IL-1β***), and *IL-8* and the concentration of reactive oxygen species (**ROS**) in the jejunum compared to the SS (*P* < 0.05). The Nano-Se and SeMet also increased the protein levels of CAT and SOD compared to the SS and Sec diet (*P* < 0.05). The number of the goblet cells and Mucin-2 (*Muc2*) levels were the highest in the Nano-Se group (*P* < 0.05). The protein expression levels of goblet cell differentiation regulator (v-myc avian myelocytomatosis viral oncogene homolog, **c-Myc**) were highest in the Nano-Se compared to the SS diet (*P* < 0.05). The Nano-Se decreased the mRNA and protein levels of NLRP3 signaling pathway-related genes compared to the SS diet (*P* < 0.05). In conclusion, our study demonstrated that Nano-Se and SeMet are better at improving the intestinal health of 21-day-old broilers. Additionally, Nano-Se demonstrated superior antioxidant and anti-inflammatory effects, promoting the development of intestinal goblet cells by modifying the NLRP3 signaling pathway.

## INTRODUCTION

The intestine plays a crucial role in the absorption, digestion, and metabolism of nutrients, significantly influencing the development of broilers (Emami et al., 2021). Ensuring the integrity of the intestinal structure is primary to achieving intestinal health.

In the poultry industry, elevated temperatures, crowded conditions, hygiene issues, and nutrient imbalances contribute to oxidative stress (Lauridsen, 2019), which can harm intestinal health and contribute to common enteric diseases like necrotic enteritis (Emami et al., 2021). Young broilers with immature intestines are more prone to enteric pathogens, reducing their resistance to oxidative stress and increasing the production of ROS, which ultimately damages the intestinal epithelium (Lauridsen, 2019).

Selenium (**Se**) is part of the enzyme glutathione peroxidase (**GPx**), prevent cellular oxidative damage and promoting the health of animals (Barchielli et al., 2022). Broilers grows rapidly and are highly susceptible to dietary Se deficiencies that lead to multiple tissue damage, like nutritional muscular dystrophy (**NMD**) (Yang et al., 2023; Zhao et al., 2023). Therefore, Sodium selenite (**SS**) has been employed for years to enhance the antioxidant content in animal feeds.

Recently, alternatives to SS, like Selenium yeast (**SY**), Selenomethionine (**SeMet**), and Selenocysteine (**Sec**), have been studied due to the high bioavailability (Briens et al., 2013; Skřivan et al., 2012). The SY alleviates cecal oxidative stress, inflammatory response, and protects the intestinal barrier from pathogens in broilers and rats (Wen et al., 2019; Yang et al., 2020).

Selenium nanoparticles (**Nano-Se**), a novel form of selenium, have garnered significant interest for adsorption capacity, low toxicity, anticancer and antimicrobial properties compared to SS (Wang et al., 2007; Sarkar et al., 2015). Nano-Se can alleviate intestinal inflammation and improve intestinal barrier caused by oxidative stress in mice (Alkhudhayri et al., 2018; Qiao et al., 2020).

Our recent study proved that Nano-Se, which was synthesized by unmodified *Bacillus subtilis* S12, could reduce oxidative stress and inflammation by down-regulating the activation of NLR family pyrin domain containing 3 (**Nlrp3**) signaling pathway, which effectively promoted intestinal goblet cells proliferation (Chen et al., 2022). However, there is limited information about the effect of various selenium sources on the intestinal health of broilers.

This study aims to examine the comparative effect of different dietary selenium sources on intestinal histology, antioxidant status, inflammatory factors, goblet cell factors, and NLRP3 signaling pathways.

## MATERIALS AND METHODS

### Animals and Experimental Design

A total of 384 one-day-old Arbor Acres broilers were divided into four dietary groups, each consisting of six replicates with 16 birds in each replicate, (half male and half female). The 4 groups were fed diets supplemental with 0.2mg/kg sodium selenite (**SS**) (control), 0.2 mg/kg Selenium nanoparticles (**Nano-Se**), 0.2 mg/kg Selenomethionine (**SeMet**), or 0.2 mg/kg Selenocysteine (**Sec**). SS (10000mg/kg) was obtained from Guangzhou Yitong Biotechnology Co., Ltd. (Guangzhou, China), SeMet (20,000 mg/kg) was obtained from Lallemand Inc. (Montreal, Canada), Sec (20,000 mg/kg) was obtained from AngelYeast Co. Ltd. (Yichang, China), and Nano-Se (30,000 mg/kg) was obtained from the Beijing Wahmix Bio-technology Co. Ltd. (Beijing, China). The basal diet (Table 1) was formulated to meet the recommended requirement of Arbor Acres Plus broilers as recommended in NY/T33-2004 (Zhang et al., 2024). The analyzed Se concentrations of the diets are listed in Table 2. The trial was performed at the Zhuozhou Poultry Research Base of China Agricultural University (Hebei, China) in Autumn, and lasted for 21 d. The starting temperature in the chicken house was adjusted to 33 ℃ and then gradually lowered to 24 ℃ by d 21. The management of birds followed the guidelines for Arbor Acres Plus broilers (Luo et al., 2023).

**Table 1:** Ingredients and nutrients analysis of the diet.

Ingredients, %	Starter diet
Corn	50.40
Soybean meal	35.40
Corn gluten meal	5.00
Corn oil	2.80
Wheat flour	2.00
Dicalcium phosphate	1.75
Limestone	1.20
Salt	0.35
Trace mineral premix^1^(No sodium selenite)	0.20
Vitamin premix^2^	0.03
Choline chloride (50%)	0.20
DL-Methionine	0.26
L-Lysine HCL	0.26
Antioxidant	0.02
Phytase	0.03
Nutrient composition, %^3^	
ME (kcal/kg)	2998
CP, %	22.89
Lysine, %	1.30
Methionine, %	0.58
Methionine+Cysteine, %	0.93
Threonine,%	0.85
Tryptophan,%	0.26
Calcium, %	0.95
NPP, %	0.40

1 The trace mineral premix provided the following per kg of diets: Cu,16 mg (as CuSO_4_·5H_2_O); Zn, 110 mg (as ZnSO_4_); Fe, 80 mg (as FeSO_4_·H_2_O); Mn, 120 mg (as MnSO_4_·H_2_O); I, 1.5 mg (as CaIO_3_); Co, 0.5 mg(CoCl_2_·H_2_O).

2 The vitamin premix provided the following per kg of diets: vitamin A, 10,000 IU; vitamin D_3_, 3,600 IU; vitamin E, 20 mg; vitamin K_3_, 2 mg; vitamin B_1_, 2 mg; vitamin B_2_, 6.4 mg; VB_6_, 3 mg; VB_12_, 0.02 mg; biotin, 0.1 mg; folic acid, 1 mg; pantothenic acid,10 mg; nicotinamide, 30 mg.

3 All the nutrient levels are calculated values.

**Table 2:** Supplemented and analyzed amount of selenium in dietary treatments (mg/kg).

	Supplemented values		Analyzed values^1^
Treatment	Sources	Levels	diets
1	Sodium selenite	0.2	0.38
2	Selenium nanoparticles	0.2	0.31
3	Selenomethionine	0.2	0.42
4	Selenocysteine	0.2	0.40

1 Selenium concentrations of dietary treatments were analyzed by hydride generation-atomic absorption spectrophotometry (Tinggi, 2003).

Nano-Se transformed from sodium selenite by the unmodified *Bacillus subtilis* S12, which fermented to convert sodium selenite into red Nano-Se with a diameter of 170 nm. The proportion of Nano-Se was 98%, and supplemental with starch into SeNPs premix, selenium content was 3694 mg/kg.

### Sample Collection

On d 21, 6 birds (one bird of approximately average weight per cage) were selected and electrically stunned. The middle sections of jejunum (approximately 1 cm) were excised for morphological analysis. Three pieces of the jejunum samples (around the middle) were also immediately collected, flushed with PBS, frozen in liquid N_2_ and then stored at –80°C for subsequent RNA and protein analysis.

### The Histological Characteristics of Jejunum Tissues

The intestinal tissues were preserved in 10% buffered formaldehyde and embedded in paraffin wax. Subsequently, 5-μm sections of each sample were stained using Periodic acid-Schiff (**PAS**) staining. The slides were examined with a Leica microscope (DMI803250593, Heidelberg, Germany) to analyze the morphology of the jejunum and the density of goblet cells. Intestinal morphological features such as villus height (**VH**), crypt depth (**CD**), and goblet cell density (per 100 um) were determined by randomly selecting 10 intact villi for each bird, following a method described in a previous study (Zhang et al., 2022).

### Validation of Differentially Expressed Gene by Quantitative RT-PCR

Total RNA was extracted from jejunum using an RNA extraction kit (9109, TaKaRa, Tokyo, Japan). The extracted RNA concentration and purity were determined using a NanoDrop 2,000 spectrophotometer (Thermo Fisher, Waltham, MA) at 260/280 nm in a range of 1.8 to 2.0. First-strand cDNA was synthesized using PrimeScript RT reagent Kit (RR047A, TaKaRa) according to the manufacturers’ instructions. Gene-specific primers of the glyceraldehyde-3-phosphate dehydrogenase (***GAPDH***), *claudin-1, claudin-3*, Occludin, zonula occluden-1 (***ZO-1***), zonula occluden-2(***ZO-2***), leucine rich repeat containing G protein-coupled receptor 5 (***Lgr5***), sucrase-isomaltase (***SI***), lysozyme (***Lyz***), chromogranin A (***Chga***), Mucin2 (***Muc2***), protein kinase B (***AKT***), Krüppel-like factor 4 (***Klf4***), SAM Pointed Domain Containing ETS Transcription Factor (***Spdef*)**, v-myc avian myelocytomatosis viral oncogene homolog (***c-Myc***), interleukin 1β (***IL-1β***), interleukin 6 (***IL-6***), interleukin 8 (***IL-8***); NLR family pyrin domain containing 3 (***Nlrp3*)**, cysteinyl aspartate specific proteinase-1 (***Caspase-1***), toll-like receptor-4 (***TLR-4***), myeloid differentiation factor88 (***Myd88***) genes were designed by the genomic data of Gallus domesticus in NCBI and synthesized by Sangon Biotech (Shanghai, China), as listed in Table 3. Real-time PCR was performed on the CFX96 Touch fluorescence quantitative PCR instrument (Bio-Rad, CA) using SYBR Green detection kit (A301-05, GenStar, Beijing, China). All the measurements were carried out in triplicate (N=6, the cage was used as an experimental unit) and the average values were calculated. The relative transcription levels of the target gene were calculated using the 2^−∆∆CT^ method, and the geometric mean of GAPDH was used to normalize the expression of the target gene.

**Table 3:** Sequence of the oligonucleotide primers used for quantitative real-time PCR.

Gene1	Primer sequence (5′→3′)	Genebank accession
*ZO-1*	F:CTTCAGGTGTTTCTCTTCCTCCTC	XM_413773
	R:CTGTGGTTTCATGGCTGGATC	
*ZO-2*	F:TGTCTGCGTGGTTGTTCCAT	XM_025144668.2
	R:CACTCACAAGGAGACGGCAG	
*Occludin*	F:TCATCGCCTCCATCGTCTAC	NM_205128.1
	R:TCTTACTGCGCGTCTTCTGG	
*Claudin -1*	F:CTGATTGCTTCCAACCAG	NM_001013611
	R:CAGGTCAAACAGAGGTACAAG	
*Claudin -3*	F:GGACACCATGTCTATGGGGC	NM_204202.1
	R:TCACGATGTTGTTGCCGATG	
*Lgr5*	F:TCAATACCTGAGCGTGCGTT	XM_425441
	R:TGTGAGTGTCAAACTCTCCAGAC	
*Muc2*	F:TCACCCTGCATGGATACTTGCTCA	NM_001318434.1
	R:TGTCCATCTGCCTGAATCACAGGT	
*Chga*	F:GCTATCTCCCTTCCTGTGACAAATG	XM_421330
	R:TGAGTTCTCTCATTGGCACCTTG	
*Lyz*	F:TACAGCCTGGGAAACTGGGT	NM_205281
	R:CTCCCATCGGTGTTACGGTT	
*SI*	F:GTACGCTACGCTTGGAGGTT	XM_015291762
	R:TGAAGAGTCACATCCATCGCAT	
*AKT*	F:CACGCTGACAGAAAACCGTG	NM_205055.1
	R: AACAACTCCCCTCCGTTAGC	
*Klf4*	F:TCAAGGCACACCTGAGAACC	XM_004949369.4
	R:GCCCGTGTGTTTTCGGTAAT	
*Spdef*	F: CTATGGGGCATCCGCAAGAA	XM_040653030.1
	R: ACAAACTGGTAGACGAGGCG	
*c-Myc*	F:ACACAACTACGCTGCTCCTC	NM_001030952.1
	R:CTCCTCTGAGTCTGACGTGC	
*IL-1β*	F:TCTGCCTGCAGAAGAAGCC	NM_204524.1
	R:CTCCGCAGCAGTTTGGTCAT	
*IL-6*	F:CAAGAAGTTCACCGTGTGCG	NM_204628.1
	R:GGAGAGCTTCGTCAGGCATT	
*IL-8*	F:GCCAAGGCTCAGCTCAATTC	NM_205498.1
	R:GCCAAGGCTCAGCTCAATTC	
*Nlrp3*	F:TGGTGTGAGGATGCTCTGTG	NM_001348947.1
	R:GACAGGTCCAGCTCCTCCA	
*Caspase-1*	F:CTGCCGTGGAGACAACATAG	XM_015295935.1
	R:AGGAGACAGTATCAGGCGTGGAAG	
*IL-18*	F:TGATGAGCTGGAATGCGATG	NM_204608.2
	R:ACTGCCAGATTTCACCTCCTG	
*TLR4*	F:CCACTATTCGGTTGGTGGAC	NM_001030693.1
	R:ACAGCTTCTCAGCAGGCAAT	
*Myd88*	F:CCGTATGGGCATGGAACAGA	NM_001030962.4
	R:CTGGCAAGACATCCCGATCA	
*GADPH*	F:ATGGCATCCAAGGAGTGAGC	NM_204305.1
	R:GGGAACAGAACTGGCCTCTC	

^1^F: forward primer; R: reverse primer.

Note: glyceraldehyde-3-phosphate dehydrogenase ***GAPDH***), Occludin, zonula occluden-1 (***ZO-1***), zonula occluden-2 ***ZO-2***), leucine rich repeat containing G protein-coupled receptor 5 (***Lgr5***), sucrase-isomaltase (***SI***), lysozyme (***Lyz***), chromogranin A (***Chga***), Mucin2 (***Muc2***), protein kinase B (***AKT***), Krüppel-like factor 4 (***Klf4***), SAM Pointed Domain Containing ETS Transcription Factor (***Spdef*)**, v-myc avian myelocytomatosis viral oncogene homolog (***c-Myc***); interleukin 1β (***IL-1β***), interleukin 6 (***IL-6***), interleukin 8 (***IL-8***), NLR family pyrin domain containing 3 (***Nlrp3*)**, cysteinyl aspartate specific proteinase-1 (***Caspase-1***), toll-like receptor-4 (***TLR-4***), myeloid differentiation factor88 (***Myd88***).

### Measurements of the Goblet Cell Differentiation Factors, Inflammatory Factors and Pyroptosis Protein by Western Blotting

Jejunum tissue (about 50 mg) extracts were homogenized with the RIPA buffer. The supernatant was collected. Proteins were separated by 10% SDS-PAGE gel and were transferred onto PVDF membranes (IPVH00010, Millipore, MA) at 200 mA for 60 min. The following antibodies were used in this experiment: Anti-c-Myc Mouse mAb (1:1,000 dilution, PTM-5028, Jingjie PTM BioLab, Hangzhou, China); SOD1 Polyclonal antibody (1:1,000 dilution, 10269-1-AP, Proteintech Group, Inc, Wuhan, China); Catalase Polyclonal antibody (1:1000 dilution, 21260-1-AP, Proteintech Group, Inc, Wuhan, China); NLRP3 Rabbit pAb (1:1,000 dilution, A12694, ABclonal, Wuhan, China); Caspase 1 Antibody (1:1,000 dilution, AF5418, Affinity Biosciences, Liyang, China); GAPDH Polyclonal antibody (1:4,000 dilution, 10494-1-AP, Proteintech Group, Inc, Wuhan, China); and Anti-beta Actin Rabbit mAb (1:1000 dilution, PTM-5143, Jingjie PTM BioLab) at 4°C overnight. At last, the blots were visualized by the BeyoECL Plus (P0018S, Beyotime, Shanghai, China). The Western blotting images were analyzed using ImageJ software (National Institutes of Health, Bethesda, MD).

### Measurements of Reactive Oxygen Species in Jejunum Tissue by ELISA

The jejunum tissue was homogenized with PBS on ice, centrifuged at 845 × *g* for 15 min, and the supernatant was collected. The concentration of ROS was measured using the Chicken reactive oxygen species (ROS) ELISA Kit (MM-6012001, Jiangsu MEIMIAN, Yancheng, China). Ultimately, the concentration was standardized based on the weight of the jejunum sample and expressed as pg/mL of tissue.

### Statistical Analysis

Statistical analysis of the data was conducted using one-way analysis of variance (ANOVA) with SPSS 20.0 software (SPSS Inc. Chicago, IL). The homogeneity of variances among all data was assessed using Levene's test. Significance among the groups was determined using the Duncan test for multiple comparisons. The results were expressed as means ± SEM, and significance was acknowledged at *P* < 0.05.

## RESULTS

### Effects of Different Se Sources on Jejunum Morphology

**Figure 1:**
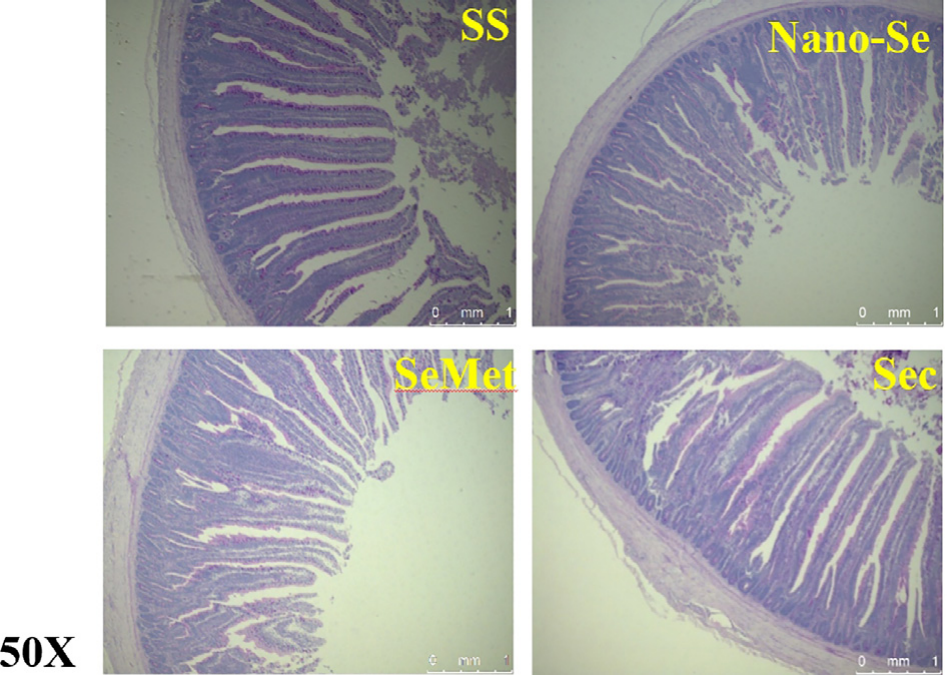
Effects of different Se sources on Jejunal morphology by the PAS method. Abbreviations: SS, Sodium Selenite; Nano-Se, Selenium nanoparticles; SeMet, selenomethionine; Sec, selenocysteine.

The change of intestinal morphology was related to the development of the intestine. Compared with the SS, dietary supplemental Nano-Se, SeMet, and Sec significantly increased intestinal VH in broilers (*P* < 0.05), with no difference among the treatments (Table 4, Figure 1). Similarly, supplementation of Nano-Se and SeMet significantly increased intestinal VH/CD ratio compared to the SS diet (*P* < 0.05), with no notable differences between Sec and SS (*P* >0.05).

**Table 4:** Effects of different Se sources on jejunum morphology of broilers.

Items	SS	Nano-Se	SeMet	Sec	SEM	*P*-value
VH (μm)	1594.75^b^	1930.5^a^	1925.5^a^	1923.4^a^	52.086	0.042
CD (μm)	200.78	191.53	186.45	195.74	3.743	0.603
VH/CD	8.04^b^	10.13^a^	10.3^a^	9.84^ab^	0.291	0.011

Abbreviations: VH, villus height; CD, crypt depth; VH/CD, villus height/ crypt depth ratio; SS, Sodium Selenite; Nano-Se, Selenium nanoparticles; SeMet, selenomethionine; Sec, selenocysteine.

Values are represented as the means ± SEM (n = 6). Means in the same row with different letters are significantly different (*P* < 0.05).

### Effects of Different Se Sources on mRNA Levels of Jejunal Barrier Protein-Related Genes

**Figure 2:**
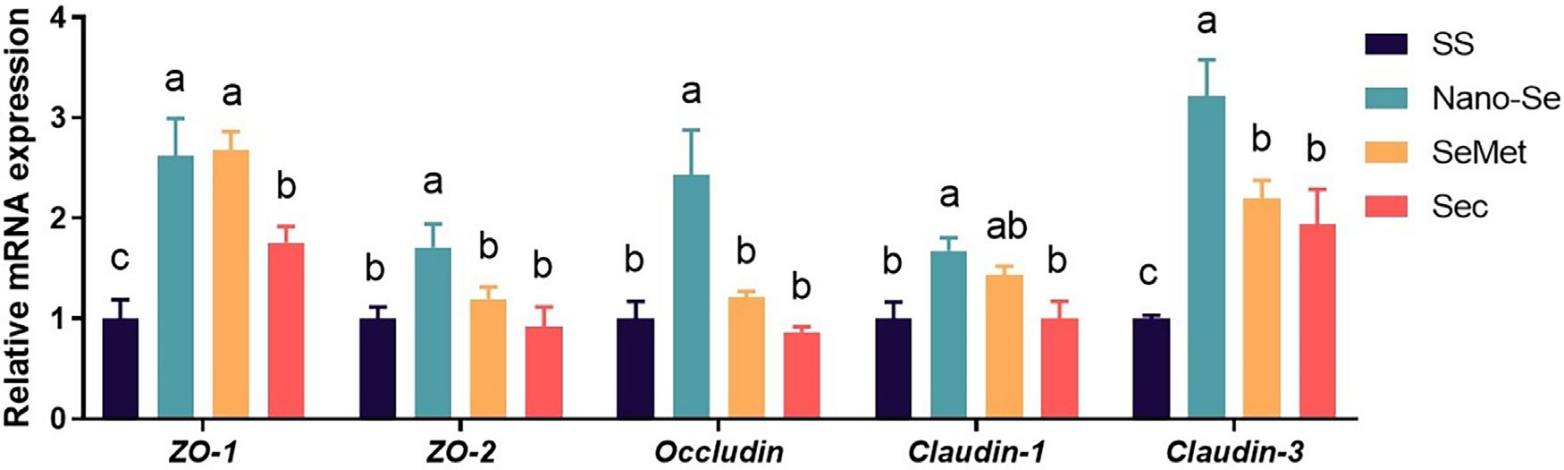
Effects of different Se sources on the mRNA expression of jejunal barrier protein-related genes of broilers. Values are represented as the means ± SEM (n = 6). Means in the same row with different letters are significantly different (*P* < 0.05). Abbreviations: SS, Sodium Selenite; Nano-Se, Selenium nanoparticles; SeMet, selenomethionine; Sec, selenocysteine.

The intestinal junction (**TJ**) is involved in the formation and functional integrity of the intestinal barrier. Adding Nano-Se and SeMet to the diets increased the mRNA levels of *ZO-1* compared to Sec (*P* <0.05), and Sec increased the mRNA levels of *ZO-1* compared to SS, with no difference between Nano-Se and SeMet (*P* > 0.05) (Figure 2).

Adding Nano-Se increased the mRNA levels of *ZO-2* and *Occludin* (*P* < 0.05) compared to SS, SeMet, and Sec, with no difference among SS, SeMet, and Sec (*P* > 0.05). Similarly, adding Nano-Se significantly increase the levels expression of *Claudin-1* (*P* < 0.05) compared to SS and Sec, and there was no significant difference between Sec and SS (*P* > 0.05).

Adding Nano-Se elevated the mRNA levels of *Claudin-3* compared to SeMet and Sec, and were significantly higher than SS (*P* < 0.01).

### Effects of Different Se Sources on Intestinal Cells

**Figure 3:**
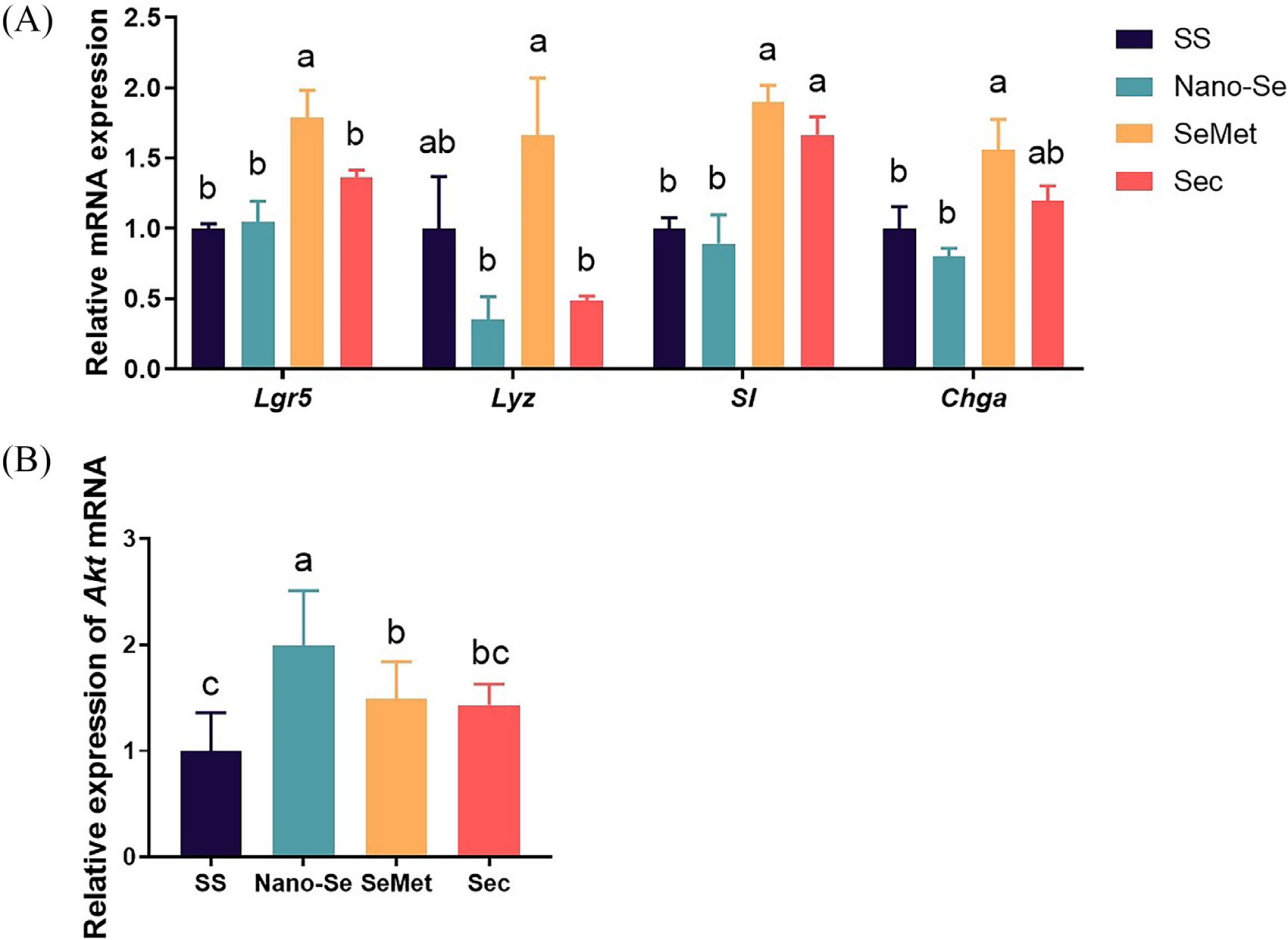
Effects of different Se sources on the expression of maker genes in jejunal cells. (A) Intestinal marker genes. (B) Intestinal proliferation signaling pathway-related genes. Values are represented as the means ± SEM (n = 6). Means in the same row with different letters are significantly different (*P* < 0.05). Abbreviations: SS, Sodium Selenite; Nano-Se, Selenium nanoparticles; SeMet, selenomethionine; Sec, selenocysteine.

Changes in intestinal morphology as described above were related to intestinal cell proliferation. The supplementation of SeMet into the diets increased the mRNA levels of *Lgr5* compared to SS, Nano-Se, and Sec (*P* < 0.01) (Figure 3A), with no difference among SS, Nano-Se, and Sec (*P* > 0.05). Supplementation of SeMet into the diets increased the mRNA levels of *Chga* compared to SS and Nano-Se (*P* < 0.01) (Figure 3A), with no significant difference between SS and Nano-Se (*P* > 0.05). Likewise, the inclusion of SeMet and Sec into the diets increased the mRNA levels of *SI* compared to SS (*P* < 0.01) (Figure 3A), with no significant difference between SeMet and Sec (*P* > 0.05). Dietary addition of SeMet sources increased the mRNA levels of *Lyz* compared to Nano-Se and Sec (*P* < 0.05) (Figure 3A), with no significant difference between Nano-Se and Sec (*P* > 0.05). In addition, the supplementation of Nano-Se increased the mRNA levels of *AKT* compared to SeMet, which was significantly greater than SS (*P* < 0.01) (Figure 3B).

### Effects of Different Se Sources on Goblet Cells of Jejunum

**Figure 4:**
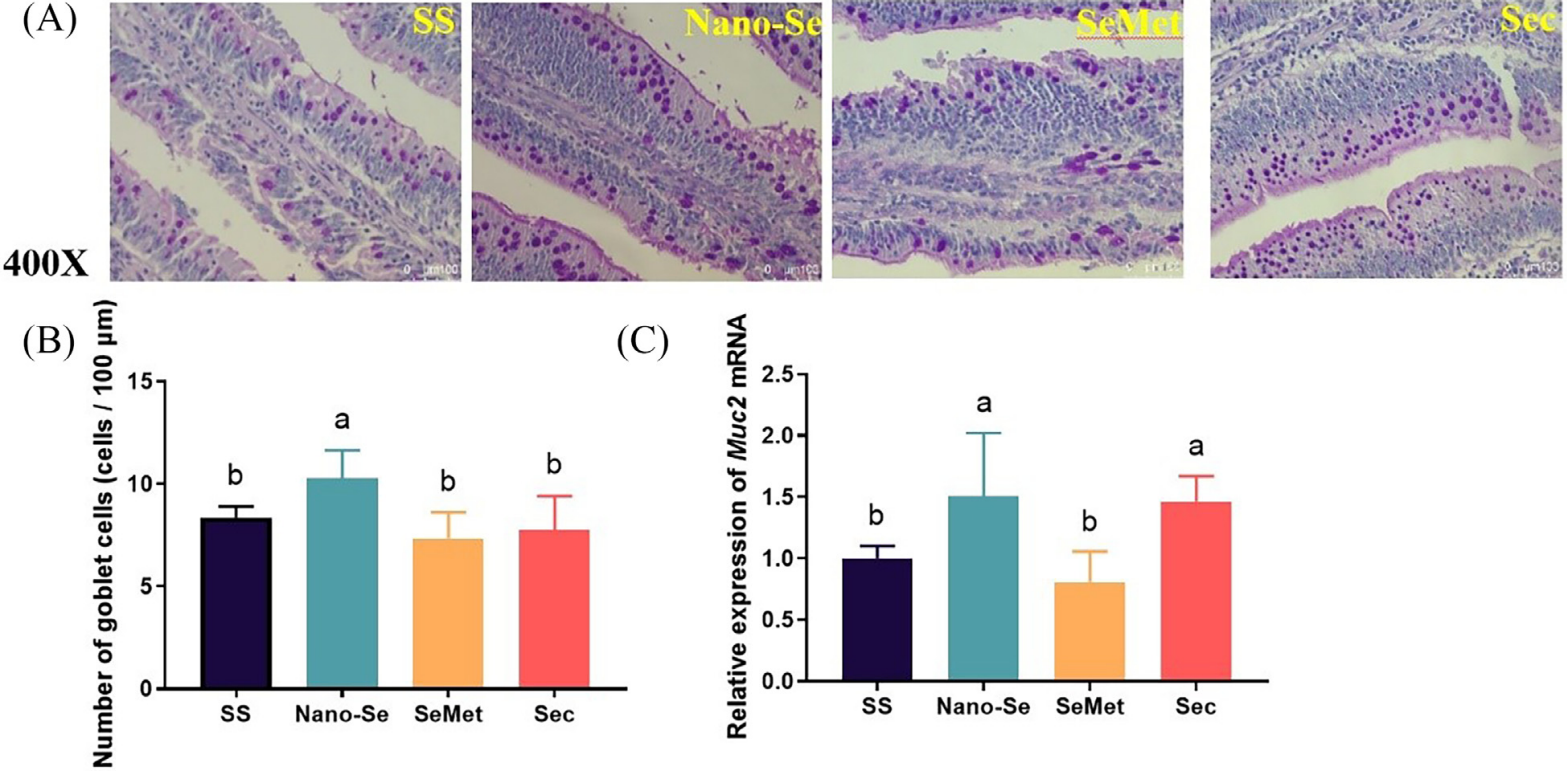
Effects of different Se sources on goblet cells of jejunum. (A) The staining of goblet cells by the PAS method. (B) The number of goblet cells in different groups. (C) mRNA levels of Muc2 in jejunum analyzed by real-time qPCR. Values are represented as the mean ± SE (n = 6). Means in the same row with different letters are significantly different (*P* < 0.05). Abbreviations: SS, Sodium Selenite; Nano-Se, Selenium nanoparticles; SeMet, selenomethionine; Sec, selenocysteine.

Goblet cells could guard the intestine against bacterial intruders. Among the Se sources, the diets with Nano-Se had the highest number of intestinal goblet cells (*P* < 0.01) (Figures 4A and 4B). The intestinal *Muc2* mRNA levels were also the highest in Nano-Se and Sec (*P* < 0.01) (Figure 4C), with no significant differences between Nano-Se and Sec (*P* > 0.05).

### Effects of Different Se Sources on Goblet Cell Differentiation Regulator of Jejunal

**Figure 5:**
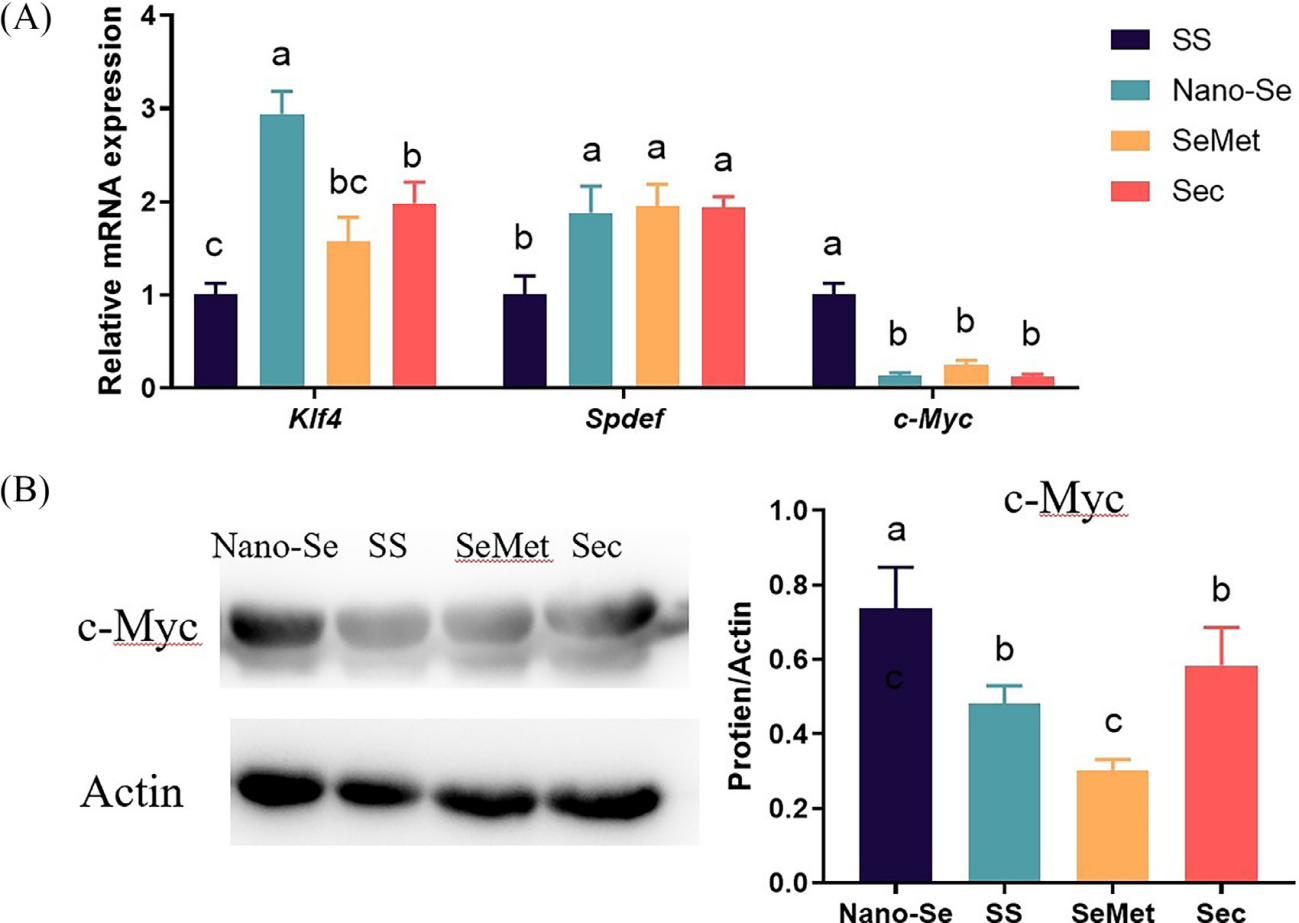
Effects of different Se sources on goblet cell differentiation regulator of jejunal. (A) mRNA levels of goblet cell differentiation regulator analyzed by real-time qPCR (n **=** 6). (B) The protein expression of c-Myc by western blot (n **≥** 3). Values are represented as the mean ± SE. Means in the same row with different letters are significantly different (*P* < 0.05). Abbreviations: SS, Sodium Selenite; Nano-Se, Selenium nanoparticles; SeMet, selenomethionine; Sec, selenocysteine.

Supplementation of Nano-Se increased the mRNA levels of *Klf4* compared to Sec, which was significantly greater than SS (*P* < 0.01) (Figure 5A).

The inclusion of Nano-Se, SeMet, and Sec into the diets increased the mRNA levels of *Spdef* compared to SS (*P* < 0.01), with no difference among the last three (*P* > 0.05).

Supplementation of Nano-Se, SeMet, and Sec decreased the mRNA levels of *c-Myc* compared to SS (*P* < 0.01), and there was no difference among the last three (*P* > 0.05). In contrast, supplementation of Nano-Se increased the protein levels of c-Myc compared to SS and Sec, which are significantly greater than SeMet (*P* < 0.01) (Figure 5B).

### Effects of Different Se Sources on the Inflammation and Oxidative Stress of Jejunal

**Figure 6:**
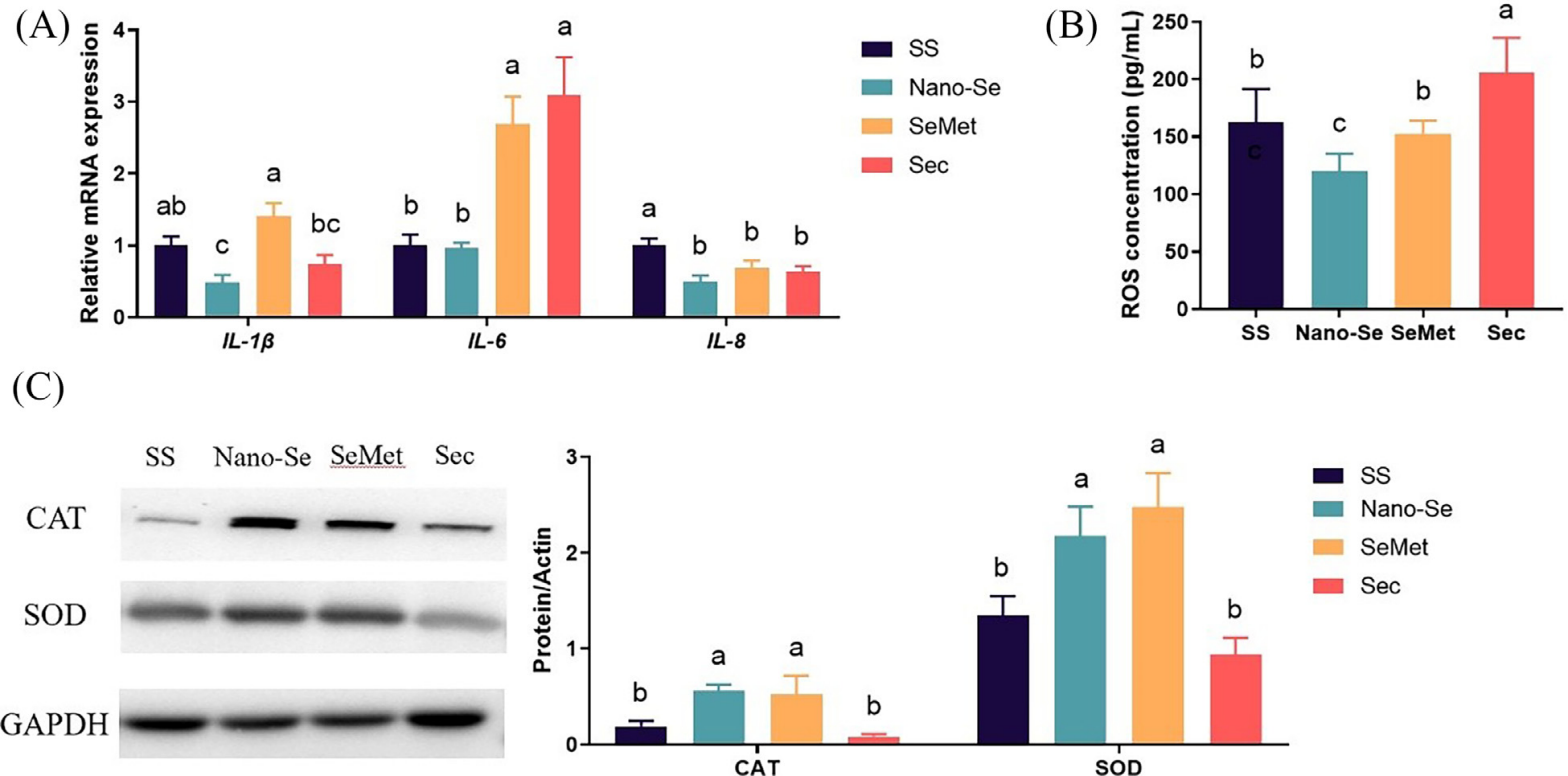
Effects of different Se sources on the inflammation and oxidative stress of jejunal. (A) mRNA levels of inflammatory cytokines analyzed by real-time qPCR (n = 6). (B) the concentration of ROS by *ELISA* (n = 6). (C)The protein expression of CAT and SOD by western blot (n ≥ 3). Values are represented as the means ± SEM. Means in the same row with different letters are significantly different (*P* < 0.05). Abbreviations: SS, Sodium Selenite; Nano-Se, Selenium nanoparticles; SeMet, selenomethionine; Sec, selenocysteine.

The inflammation and oxidative stress of the intestinal mucosa play critical roles in responding to infectious pathogens. Adding Nano-Se decreased the mRNA levels of *IL-1β* (*P* < 0.05) compared to SS and SeMet (*P* < 0.01) (Figure 6A), with no difference among SS and SeMet (*P* > 0.05). The inclusion of Nano-Se, SeMet, and Sec into the diets decreased the mRNA levels of *IL-8* compared to SS (*P* < 0.01), with no difference among the last three (*P* > 0.05). In contrast, supplementation of SeMet and Sec increased the mRNA levels of *IL-6* compared to the SS and Nano-Se diet (*P* < 0.01).

The concentration of ROS was significantly (*P* < 0.01) lower with supplementation of Nano-Se and was higher (*P* < 0.01) with supplementation of Sec than chicks fed the SS diet (Figure 6B). Similarly, supplementation of Nano-Se and SeMet increased the protein levels of CAT and SOD compared to the SS and Sec diet (*P *< 0.05, Figure 6C).

### Effects of Different Se Sources on Pyroptosis-Related Genes

**Figure 7:**
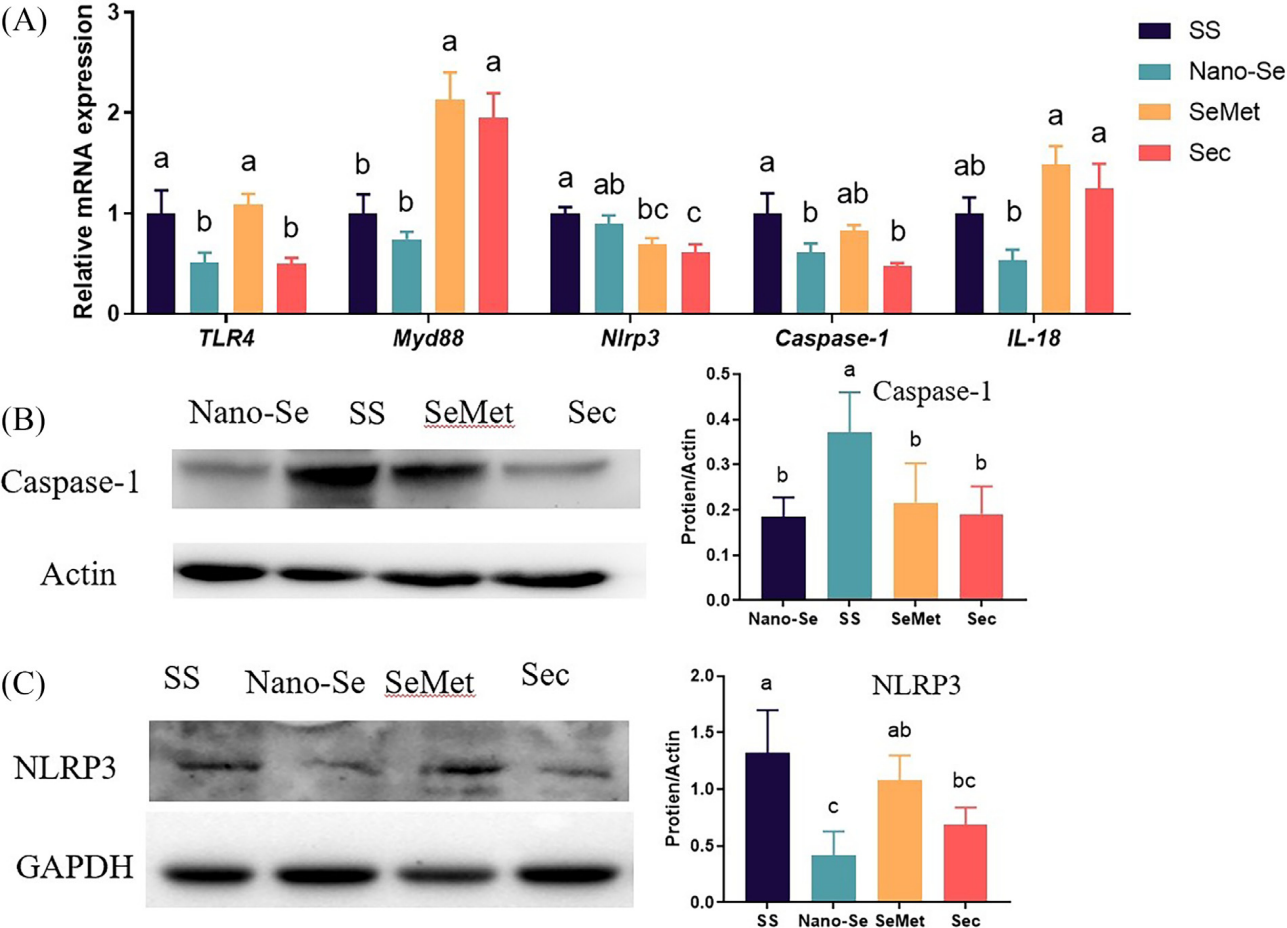
Effects of different Se sources on pyroptosis-related genes. (A) mRNA levels of pyroptosis-related genes analyzed by real-time qPCR (n = 6). (B and C) the protein expression of pyroptosis-related genes by western blot (n ≥ 3). Values are represented as the means ± SEM. Means in the same row with different letters are significantly different (*P* < 0.05). Abbreviations: SS, Sodium Selenite; Nano-Se, Selenium nanoparticles; SeMet, selenomethionine; Sec, selenocysteine.

The change of *IL-1β* mRNA levels indicated that pyroptosis was involved in intestinal development. Supplementation of Nano-Se and Sec decreased the mRNA levels of *TLR4* (*P* < 0.01) and *Casepase-1* (*P* < 0.05) compared to the SS diet (Figure 7A), and there was no significant difference between Nano-Se and Sec (*P* > 0.05). Supplementation of Nano-Se decreased the mRNA levels of *IL-18* compared to SeMet and Sec (*P* < 0.01), with no significant difference between SeMet and Sec (*P* > 0.05). Supplementation of SeMet and Sec decreased the mRNA levels of *Nlrp3* compared to the SS diet (*P* < 0.01), with no notable differences between SeMet and Sec (*P* > 0.05). In contrast, supplementation of SeMet and Sec in the poultry diet increased the mRNA levels of *Myd88* compared to SS and Nano-Se (*P* < 0.01).

Supplementation of Nano-Se, SeMet, and Sec decreased the protein levels of Casepase-1 compared to the SS diet (*P* < 0.01) (Figure 7B), and there was no difference among Nano-Se, SeMet, and Sec (*P* > 0.05). Supplementation of Nano-Se and Sec decreased the protein levels of NLRP3 compared to the SS diet (*P* < 0.05, Figure 7C).

## DISCUSSION

**Figure 8:**
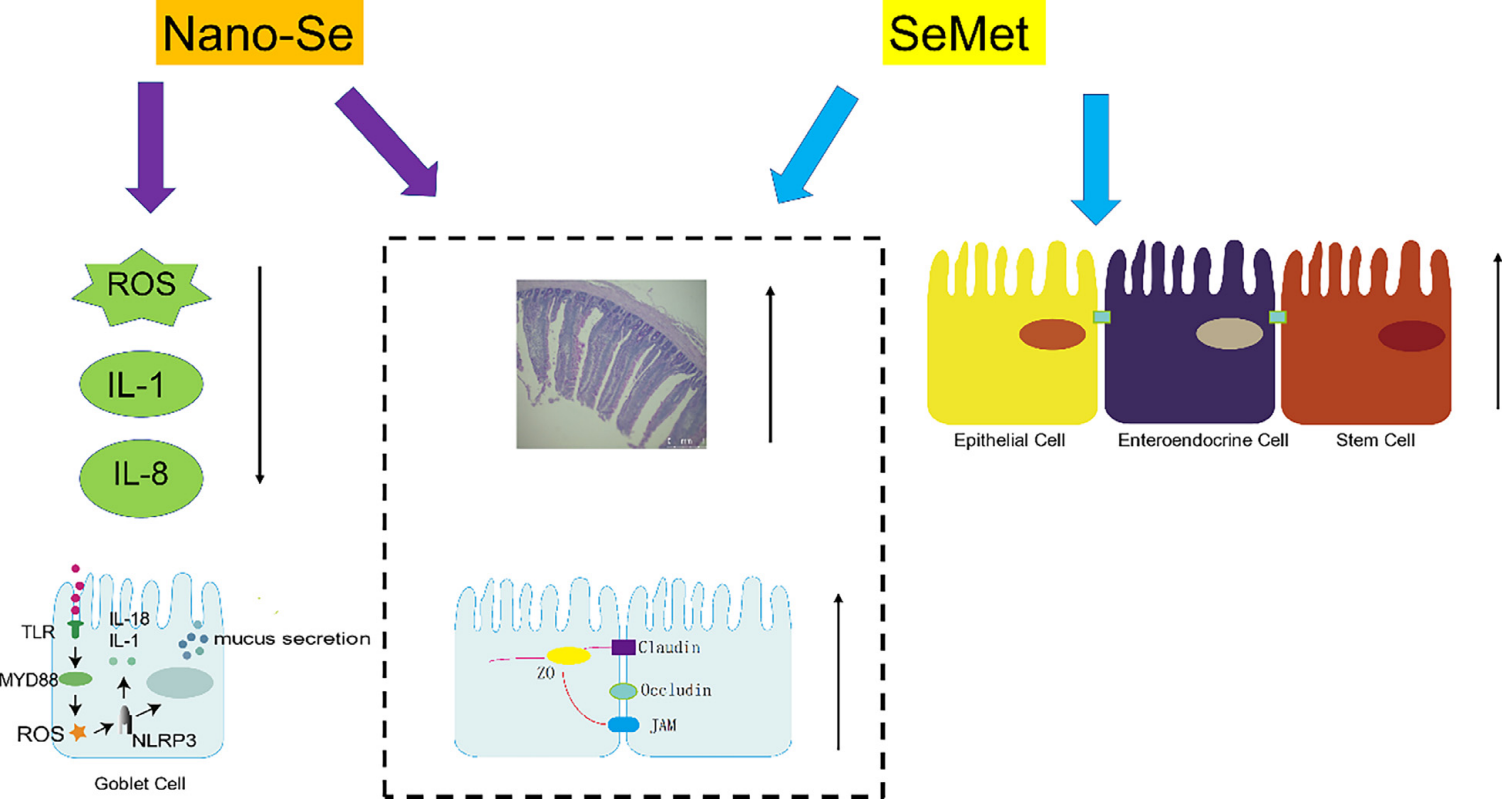
Model of Nano-Se and SeMet promoting intestinal health in broilers.

Despite growing evidence highlighting the significance of Se in broiler growth, the influence of different selenium sources on intestinal health is still unclear. Here, we successfully found that the Nano-Se and SeMet were more effective than the SS in improving gut health in broiler. Nano-Se has better antioxidant and anti-inflammatory abilities to promote the development of intestinal goblet cell by inhibiting NLRP3 signaling pathway(Figure 8).

The present study revealed showed that dietary Nano-Se and SeMet supplementation significantly increased intestinal VH and VH/CD in broilers compared to the SS diet. Changes in intestinal morphology are linked to factors such as intestinal development, surface area, secretory cell count, inflammation, and antioxidant activity (Tang et al., 2020). The TJ includes transmembrane and peripheral proteins (occludin, claudin, and ZO), serving as vital barriers to regulate intestinal cell permeability and uphold junctional structural integrity (Li and Ajuwon, 2021). The study revealed that adding Nano-Se to the diets increased the mRNA levels of *ZO-1, ZO-2, Occludin, Claudi*n-1, and *Claudin-3*. The SeMet supplementation raised the expression of ZO-1 and Claudin-3. Upregulating the expression of TJ proteins enhanced intestinal integrity, reducing para-cellular permeability, forming a barrier against pathogen entry, and resisting oxidative stress (Eichner et al., 2017; Tan et al., 2018). Thus, Nano-Se and SeMet could enhance intestinal morphology, facilitating intestinal development.

Elevated VH/CD ratios suggest a greater need for cell proliferation to uphold intestinal barrier integrit (Awad et al., 2011). The Se may delay apoptosis and enhance enterocyte viability by regulating inflammatory cytokine activity and boosting antioxidant status (Ahmadipour et al., 2015; Safdari-Rostamabad et al., 2017). In this study, SeMet increased the marker genes for intestinal enteroendocrine cells (***Chga***), stem cells (***Lgr5***), and epithelial cells (**SI**). The inclusion of Nano-Se and SeMet also increased the levels of *AKT*. Xiao et al. (2019) previously discovered that Nano-Se stimulated intestinal epithelial cell proliferation through the FXR-PI3K/AKT signaling pathway, which supports our result. The enhancement of intestinal structure may be linked to the role of Nano-Se and SeMet, positively impacting enterocyte viability through Se's active contribution in selenium-containing proteins (Dalia et al., 2020).

Inflammatory cytokines in the intestine can respond to infectious pathogens (Shirley and Lillehoj, 2012). Research indicates that selenium and SS elevate intestinal levels of IL-1, IL-6, and IL-8, strengthening immune responses against avian pathogens (Lee et al., 2014; Yang et al., 2020). Dietary Se insufficiency caused increased levels of IL-1β and IL-6 in chicks and induced inflammatory injury in the bursa of fabricius and the heart (Bai et al., 2022; Ji et al., 2024). In this study, Nano-Se lowered the mRNA levels of *IL-1β* and *IL-8* in the jejunum compared. Conversely, SeMet and Sec raised the mRNA levels of *IL-6* compared to the SS and Nano-Se diet. Intestinal cells experience oxidative damage, leading to cellular apoptosis via mitochondria-dependent and mitochondria-independent pathways (Sinha et al., 2013). Selenium deficiency induced apoptosis of chicken intestinal cells through an inflammatory signaling-induced death receptor pathway (Wang et al., 2018), which can damage intestinal tissues (Yu et al., 2015). Selenium enhances the antioxidant capacity of chickens and activates antioxidant enzymes (GPX and TrxR), reducing the production of ROS (Labunskyy et al., 2014). Our study proved that Nano-Se and SeMet increased the protein levels of CAT and SOD, which could reduce ROS production. Previous studies demonstrated that Nano-Se and higher doses of organic Se could improve the antioxidant capacity of tissues (Bakhshalinejad et al., 2018; Li et al., 2018). The current study found that the level of ROS was lowered by supplementation of Nano-Se and was higher by supplementation of Sec. Therefore, compared with other Se sources, Nano-Se can reduce intestinal ROS production and decrease the inflammatory response, by increasing the antioxidant enzymes activity like SOD and CAT.

The Muc2 is synthesized by goblet cells (Birchenough et al., 2015). The growth of intestinal goblet cells was dependent on ROS production and the level of inflammatory factors (IL-4 and IL-13) (Birchenough et al., 2015; Gehart and Clevers, 2019). Increased levels of IL-4 and IL-13 promoted the differentiation and proliferation of goblet cells through the STAT6 signaling pathway (Oeser et al., 2015). The Nano-Se greatly enhanced goblet cell density and improved mucin structure in the broiler jejunum (Alkhudhayri et al., 2018; Bami et al., 2022; Wen et al., 2019). Similarly, this experiment showed that the number of intestinal goblet cells and Muc2 levels were the highest in the Nano-Se group. The Klf4, Spdef, and c-Myc are vital for the growth and maturation of goblet cells and control genes related to mucus production (Noah et al., 2010; Konsavage et al., 2012; Katano et al., 2020). In this study, Nano-Se increased the mRNA expression of *Spdef* and *Klf4*, which could support the enhancement of goblet cell differentiation. Adding Nano-Se increased the c-Myc protein expression, indicating a significant promotion of goblet cell differentiation. These results demonstrated that Nano-Se effectively boosts the number and differentiation of intestinal goblet cells.

Growing evidence indicates that the excess production of ROS, and pathogens can lead to inflammation and cell death (Haghi-Aminjan et al., 2018; Frank and Vince, 2019). There is substantial evidence that pyroptosis plays a role in protecting hosts from pathogenic infections (Frank and Vince, 2019). The NLRP3 inflammasome serves as an inflammation mediator (Takahashi, 2014) and is implicated in various gut-related diseases (Donovan et al., 2020). The NLRP3/caspase-1/IL-1 pathway is a key inflammatory pathway activated by elevated ROS (Sahin et al., 2019). When activated, NLRP3, ASC, and pro-caspase-1 bind to form an active caspase-1, which cleaves other cytosolic targets (IL-1 and IL-18), initiating inflammatory responses (Schneider et al., 2017). Some NLRPs are found in intestinal cells, suggesting their role in intestinal homeostasis (Wlodarska et al., 2014). Goblet cells nonspecifically endocytose TLR ligands, activating the NLRP6 signaling pathways (Birchenough et al., 2016), promote Muc2 secretion in goblet cells and enhance the formation of mucus layers (Wlodarska et al., 2014; Volk et al., 2019). In the present study, supplementation of Nano-Se decreased the mRNA levels of *IL-18, TLR4*, and *Casepase-1.* The Supplementation of SeMet decreased the *Nlrp3* expression. Furthermore, NLRP3 participated in selenium's antagonism against lead-induced inflammation by modifying oxidative stress and inflammatory genes in chicken (Huang et al., 2021). In this study, adding Nano-Se and Sec decreased the protein expression level of NLRP3 and Casepase-1 compared to the SS diet.

## CONCLUSIONS

In conclusion, Nano-Se and SeMet could improve the intestinal structure and promote the expression of tight junction proteins in 21-day-old broilers. Moreover, Nano-Se has better antioxidant and anti-inflammatory abilities to promote intestinal goblet cell development by modifying the NLRP3 signaling pathway.

## DISCLOSURES

The authors declare no conflict of interest.
